# Fall sensors, home emergency system, and social service for ≥ 75-year-olds living at home - a matched control intervention study

**DOI:** 10.1186/s12877-025-05856-2

**Published:** 2025-04-02

**Authors:** Jan C. Zoellick, Sonia Lech, Julie L. O’Sullivan, Eva Jansen, Juliana Supplieth, Ronny Kuhnert, Ulrike Grittner, Johanna Schuster

**Affiliations:** 1 Institute of Medical Sociology and Rehabilitation Science, Charité – Universitätsmedizin Berlin, corporate member of Freie Universität Berlin and Humboldt-Universität Zu Berlin, Charitéplatz 1, 10117 Berlin, Germany; 2 Department of Psychiatry and Neurosciences, Charité – Universitätsmedizin Berlin, corporate member of Freie Universität Berlin and Humboldt-Universität Zu Berlin, Charitéplatz 1, 10117 Berlin, Germany; 3 Institute of Biometry and Clinical Epidemiology, Charité – Universitätsmedizin Berlin, corporate member of Freie Universität Berlin and Humboldt-Universität Zu Berlin, Charitéplatz 1, 10117 Berlin, Germany

**Keywords:** Emergency prevention, Home emergency system, Intervention, Older adults

## Abstract

**Background:**

Medical emergencies occur frequently at home and during leisure activities. Digital technologies hold great potential for novel approaches towards emergency detection and treatment. The purpose of this study was to evaluate an integrated home-based emergency call system.

**Methods:**

We conducted a matched-control intervention study with 180 participants (*M*: 81.7 years; *SD*: 4.1 years; 68% female) in the intervention group (IG) and 708 matched controls (*M*: 81.4 years; *SD*: 3.9 years; 68% female). The intervention targeted ≥ 75-year-old community-dwelling adults and consisted of a base station, motion sensors for the home, a necklace with fall detection sensors, and a social service. We expected fewer emergency contacts and fewer hospitalisations in the IG than in the control group (CG). Secondary outcomes were healthcare costs and subjective assessments by participants. Negative binomial regression models and linear regression analyses were used to test the primary and secondary hypotheses.

**Results:**

Our results revealed similar rates of emergency contacts (IRR = 0.89 [95%-CI:0.62–1.28]; *p* = .523) and hospitalisations (IRR = 1.23 [95%-CI:0.95–1.60]; *p* = .122) with similar durations (M_Diff_ = -2.79 days [95%-CI:-7.63–2.06]; *p* = .260) and similar healthcare costs (-7%, [95%-CI: -54%-40%], p = .774) in the IG compared to matched controls (intention to treat approach). Regarding changes in the IG over time, participants reported worse subjective outcomes, e.g., lower health-oriented quality of life (M_t0_ = 40.4; SD_t0_ = 8.7; M_t1_ = 37.6; SD_t1_ = 8.0; *t*(124) = -4.10; *p* ≤ .001) at the end of the 12 months intervention period compared to the beginning of the study.

**Conclusions:**

The intervention had no effect on hospitalisations and emergency contacts. However, participants made also little use of the intervention. The observed decline in subjective health and other subjective outcomes may be attributed to the high age of participants at intervention start and overall circumstances due to the COVID pandemic. The market for technologies for older adults is highly dynamic and growing quickly; thus, more suitable and effective technologies might be developed soon. These novel technologies should be evaluated accordingly before entering the market.

**Trial registration:**

DRKS00023171 (https://drks.de/search/en/trial/DRKS00023171).

**Supplementary Information:**

The online version contains supplementary material available at 10.1186/s12877-025-05856-2.

## Introduction

A medical emergency is defined as “an illness, injury, symptom or condition so serious that a reasonable person would seek care right away to avoid severe harm” [1]. For Germany and USA, the prevalence of medical emergencies is about 169 and 161 in 1,000 inhabitants, respectively [2, 3]. Most accidents and medical emergencies occur at home [3]. Even though reliable data for home emergencies are difficult to obtain, the US National Safety Council estimates that 78% of preventable injury-related deaths occur at home with *poisoning/overdosing* and *falls* as by far the most frequent reasons for injury-related deaths [3]. Studies report an annual fall prevalence of 30% in community-dwelling adults above 65 years increasing with age [4, 5]. Most falls do not require acute medical attention or lead to severe acute outcomes; however, about 10% of falls result in fractures that can consequently lead to hospitalisations, admittance to nursing homes, or premature deaths [6, 7]. Risk factors for falls include older age, the existence of chronic diseases, and being a woman [7] as well as polypharmacy and sarcopenia [8]. Previous intervention studies for fall prevention have focused mainly on exercise and home safety reducing the number of falls per person in a given time period, but not reducing the number of people falling [5]. Accordingly, recommendations include exercise trainings and advice on fall risks together with a “comprehensive multifactorial falls risk assessment with a view to co-design and implement personalised multidomain interventions” [9]. Current intervention studies aim to estimate the impacts of personalised care plans and comprehensive fall risk assessments after a fall incident [10]. Through prevention strategies, hospitalisation rates have been reduced by 7% [11]. Hospitalisations as inpatient care are resource intensive compared to outpatient treatments. Thus, reducing (longer) inpatient care in favour of outpatient treatment is resource-efficient, which is particularly important in healthcare systems with high cost pressure and in an aging society such as Germany [12, 13]. While the immediate use of low-threshold healthcare services might increase [14, 15], the goal of the interventions is to reduce the resource-intensive long-term consequences [14–16].

Technologies for fall detection include accelerometers in necklaces, watches, or floor mats that activate in the event of a fall and oftentimes trigger an emergency call automatically [17]. These sensors can be integrated into an ambient assisted living (AAL) system, i.e., connected sensors such as smart meters or motion detectors distributed in the entire living space that monitor movements and activities to create a comprehensive profile of the resident [18, 19]. Such an integrated system promises immediate responses particularly relevant for people living alone who might otherwise not receive help quickly. Technological components can be complemented with social workers that provide additional services such as information or social support [20]. Together, these socio-technological solutions can support ageing in place, directly addressing the needs and wishes of many older adults [21, 22]. However, evidence for the effectiveness of AAL systems is not conclusive, and the need for publishing null findings as informative evidence is stressed in methodological reflexions [23]. While some studies find increases in self-reported quality of life [24], others do not find effects on participant self-reports [23]. Consequently, more research is expected to emerge in the upcoming years [25].

The purpose of this study was to evaluate an integrated system for emergency detection and support consisting of motion sensors, a home emergency call system, and a social service. The two primary hypotheses stated that the intervention group (IG) shows significantly fewer medical emergency contacts and fewer hospitalisations than the matched control group (CG) within the 12 months of the intervention. The secondary hypotheses were: the IG shows reduced costs for care regarding hospital and nursing costs than the matched CG within the 12 months of the intervention; and the IG shows fewer concerns about falling and less perceived stress, as well as higher social support at follow-up (t_1_) than at baseline (t_0_).

## Methods

### Procedure

#### Intervention and study design

The aim of the intervention was to enable people over the age of 75 years to remain independent in their own homes for longer and at the same time to stabilise their health, reduce health risks, and reduce avoidable need for care, in particular emergency and hospital stays. By identifying health risks at an early stage through a low-threshold intervention, the project aimed to avoid lengthy and resource-intensive treatment. For this purpose, we targeted community-dwelling adults above the age of 75 years in and around Berlin, Germany. For the duration of 12 months, participants of the IG received eight motion sensors placed at central locations within the living space (e.g. bedroom, fridge) and a home emergency call system consisting of the base station as well as a necklace with an accelerometer and an emergency button pendant that automatically registered falls and consequently sent an emergency call. In cases of emergency, participants could also manually activate their pendant or the base station to be referred to an emergency call centre. These elements aimed to enable monitoring and to provide immediate and targeted emergency care, which could give participants a sense of security and protection [26]. Participants were also registered with a social service provider offering support in grocery shopping, phone calls with volunteers for social support, arranging services such as domestic help or transportation, nursing advice, or a locksmith. The integrated socio-technological intervention could therefore promote the mental and physical health of the participants and potentially reduce high follow-up costs by means of quick emergency care.

Upon inclusion, study participants received information on these offers from the social service provider’s employees and were able to choose from them. Participants also received explanations for the monitoring system. The participants did not have to actively use the system, only if they wanted to make an emergency call or contact the social services provider. The social service monitored participants daily via an interface showing data from the sensors and the emergency call system. If sensor data indicated irregularities, the social service provider could contact the participants to check in and aid if needed. Thus, the intervention combined technical components from classical AAL systems with a social service provider to form a complex intervention [27]. The intervention was embedded in the multifaceted German healthcare landscape with outpatient and inpatient care provision and a structural division between healthcare and nursing on an insurance level [12].

We used a matched-control study with mixed methods to test the efficacy of the intervention. We conducted telephone surveys with the IG participants at the start (t_0_) and end (t_1_) of the intervention, and we obtained health insurance data for both the IG and a matched CG for the intervention period. The project evaluation additionally included an acceptability survey amongst potentially eligible people, ten semi-structured interviews with potentially eligible people, interviews with the social service providers, and two focus groups with participants and project partners. All guidelines and surveys were designed specifically for this study, and are included in the supplementary material. In this present article, we focus on the healthcare data and telephone surveys targeting the primary and secondary hypotheses. We excluded the other data sources from this article, because these data were collected from other individuals than participants in the IG or CG, or because the data were published elsewhere already, i.e. in the case of focus groups and semi-structured interviews [28].

#### Inclusion and exclusion criteria

Inclusion criteria were being health-insured with one of three project partner health insurance companies, age ≥ 75 years, living in housing from the project partner housing company or in a predetermined set of Eastern Berlin postal codes, and a care level of 2 or less signalling minor or considerable impairments on a scale from 1 (minor impairments) to 5 (most severe impairments) [29]. Exclusion criteria were a care level above 2, no health insurance claims data, acute suicidality, living in stationary care or needing 24-h care assistance, or one of the following diagnoses H91.3, H54.4, F10, F20, F30, or F31.

#### Power calculation

Based on a previous study [11], we anticipated a difference in hospitalisation rates of 7% (7% vs. 14%) between the IG and the matched CG representing a small to medium effect size (Cohen’s *d* = 0.24) using a two-sided level of significance (α = 0.05) and power (1-β = 0.80). We originally envisioned an allocation ratio of 1:2 between IG and CG that was adapted to 1:5 in the COVID-pandemic when cautionary measures for recruiting participants were implemented. Our initial power calculation using G*Power 3.1.9.2 [30] with z-tests and differences between two independent proportions as test family revealed *N* = 186 participants and *N* = 372 controls with a recruitment target of *N* = 207 participants based on 10% dropouts. Our updated power calculation with an allocation ratio of 1:5 revealed *N* = 152 participants and *N* = 760 controls with a recruitment target of *N* = 167 participants based on 10% dropouts. The change in allocation ratio also meant fewer contacts for members of the then so-called “high-risk group for COVID” of older adults that might have resulted from participating in this study. Both primary hypotheses were tested hierarchically, meaning the first primary hypothesis (fewer emergency contacts in IG) was tested at a two-sided significance level of 5%, and if significant, the second primary hypothesis (fewer hospitalisations in IG) was tested at the same significance level. In case of no significant result for the first hypothesis, the second analysis will be exploratory. All secondary outcomes were analysed exploratorily without adjustment for multiple testing. We also report results of sub group analyses for sociodemographic variables as exploratory analyses.

#### Recruitment

Participants were invited to participate through mailings from the health insurance providers in the fourth quarter of 2020. The mailings contained written study information as well as contact details of the project team. The insurance company and the social service provider then provided further oral information about the study, and the participants signed informed consent. The three health insurance providers sent out 4,111 mailings, and they received *N* = 228 answers resulting in a response rate of 6%.

#### Matching

We compiled a matched CG from insured clients in one of the three health insurance companies corresponding to the inclusion criteria. Matching was performed regarding the most important covariates: (1) age at study initiation, (2) sex, (3) health insurance, (4) care level at study initiation, (5) number of hospitalisations in the six months prior to study initiation, (6) number of physician contacts in the six months prior to study initiation, and (7) postal code. Mahalanobis distance matching was employed, with a matching ratio of 1:4 (one patient from the IG was matched with up to 4 patients from the CG). Exact matching was carried out for sex, care level, health insurance, and hospitalisations in two quarters before t_0_ dichotomised into “yes” or “no” within the matching process, and a caliper was set at the level of the respective standard deviation for age in years (caliper = 0.7) and number of physician contacts (caliper = 3). Smaller calipers would lead to more suitable matches but fewer matches overall. Therefore, the calipers were set as described. The matching procedure was conducted using the R package MatchIt [31].

### Definitions and measures

The primary outcomes were the number of medical emergency contacts and hospitalisations within 12 months (duration of intervention) obtained via the claims data of health insurance companies. We also obtained sex (male, female, diverse) and age categories at t_0_ (75–79 years, 80–84 years, 85 + years) via claims data. We additionally analysed costs for care regarding hospital and nursing costs via claims data. Claims data include information on medical treatments, hospital stays, pharmaceuticals, and outpatient care providing a comprehensive overview of cost structures in the German healthcare system. We measured the following outcomes via self-reports from participants:

- *Falls* are “unintentional change[s] in position resulting in coming to rest on the ground or other lower level” [32]. Participants were asked to recall the number of falls in the previous 12 months.
- *Health-related quality of life* (HR-QOL) is a multi-dimensional concept, which we measured with the SF-12 [33, 34]. The scoring produces a physical component summary (PCS) and a mental component summary (MCS). Scores above 50 indicate a better-than-average HR-QOL, whereas scores below 50 suggest a below-average HR-QOL.
- *Concerns about falling* were measured using the falls efficacy scale (FES-I) [35, 36]. The scale contains 16 items measuring the perceived fall risk in different activities on 4-point scales ranging between 1 (*not at all concerned*) and 4 (*very concerned*).
- *Perceived stress* was measured using the perceived stress scale [37]. The scale contains 10 items on 5-point Likert scales ranging between 1 (*never*) and 5 (*very often*).
- *Social support* was measured using the F-SozU K-14 [38], which measures social support in the natural environment (general social support), excluding assistance from healthcare professionals. The F-SozU K-14 contains 14 statements about perceived or anticipated social support rated on 5-point Likert scales, ranging from 1 (*does not apply*) to 5 (*applies strongly*).

We measured the following covariates via self-reports from participants: *Instrumental activities of daily living* (IADL) using the Lawton IADL Scale [39, 40] that evaluates a person’s ability to perform tasks like using a telephone, doing laundry, and managing finances. Summary scores range from 0 (*low function, dependent*) to 8 (*high function, independent*).

*Activities of daily living* (ADL) refer to basic abilities that are required for self-care, such as eating or bathing [39]. We used the Barthel Index [41] to measure performance in ADL. The total possible scores range from 0 to 100, with lower scores indicating greater dependence.

*Technology commitment* (TC) was measured with the short scale for TC [44]. It contains 12 items on 5-point Likert scales ranging between 1 (*don’t agree at all*) and 5 (*agree completely*).

Lastly, we collected technical data from the emergency buttons and the sensors in the form of alarms and “risk scores” indicating irregularities. The social service coded alarms in terms of their content as “accidentally triggered”, “notification”, “test”, “technical notification”, “emergency”, “false alarm”, or “deviation in dashboard” and in terms of their trigger as “automatically” or “manually”. Additionally, the consequences of the alarms were described as “no assistance required”, “assistance: emergency control centre”, “assistance: locksmith”, or “assistance: contacted relative”.

### Analysis

We performed all analyses regarding health insurance claims data for the *intention to treat* (ITT) approach with all 180 participants who started the intervention and provided health insurance data and their respective matched controls. We also performed all health insurance claims data analyses *per protocol* (PP) with the 165 participants who underwent the full 12 months of intervention and their respective matched controls.

For descriptive analyses, we used means, standard deviations, medians, interquartile ranges, and absolute and relative frequencies.

Analyses regarding the primary outcomes were performed hierarchically with the number of emergency contacts tested first at the significance level α = 0.05, and the number of hospitalisations afterwards. To address the primary and secondary hypotheses regarding the claims data of health insurance companies, we calculated negative binomial regressions in case of count data resulting in incidence rate ratios (IRRs) and 95% confidence intervals (95%-CIs). We included all outliers in the analyses. After checking all assumptions for the models, we calculated and reported negative binomial regression-based IRRs adjusted for sex, age, care level at t_0_, and number of hospitalisations before t_0_. We chose the covariates as they are known to significantly influence health outcomes, e.g., the number or duration of hospitalisations. To assess the robustness of our findings, we conducted sensitivity analyses using a more restrictive Poisson regression model. This alternative model produced comparable results.

We calculated zero-inflated models resulting in mean/percentage differences and 95%-CIs for the duration of hospital stays and costs. Zero-inflated models can be used for data that has an excess of zero values, combining a standard model (here: linear) with a separate process that models the excess zeros. We used the model here to appropriately account for the high proportion of zeros. The costs were log-transformed to limit the influence of outliers. We added one euro for each participant to ensure the logarithm can be calculated even when there are no costs.

To account for the matching assignment, Generalized Linear Mixed-Effects Models were applied. The matching ID was used as a random intercept to represent the assignment of IG to the matched controls. All results were calculated in R (version 4.3.1). The function glmer was used for linear and Poisson regression and glmer.nb for negative binomial regression. Both functions are provided by the lme4 package [43]. The package glmmTMB was used for the zero-inflated models [44]. Lastly, we calculated dependent sample *t*-tests for participants in the IG regarding differences in their self-reported data between t_0_ and t_1_.

## Results

### Sample

**Fig. 1:**
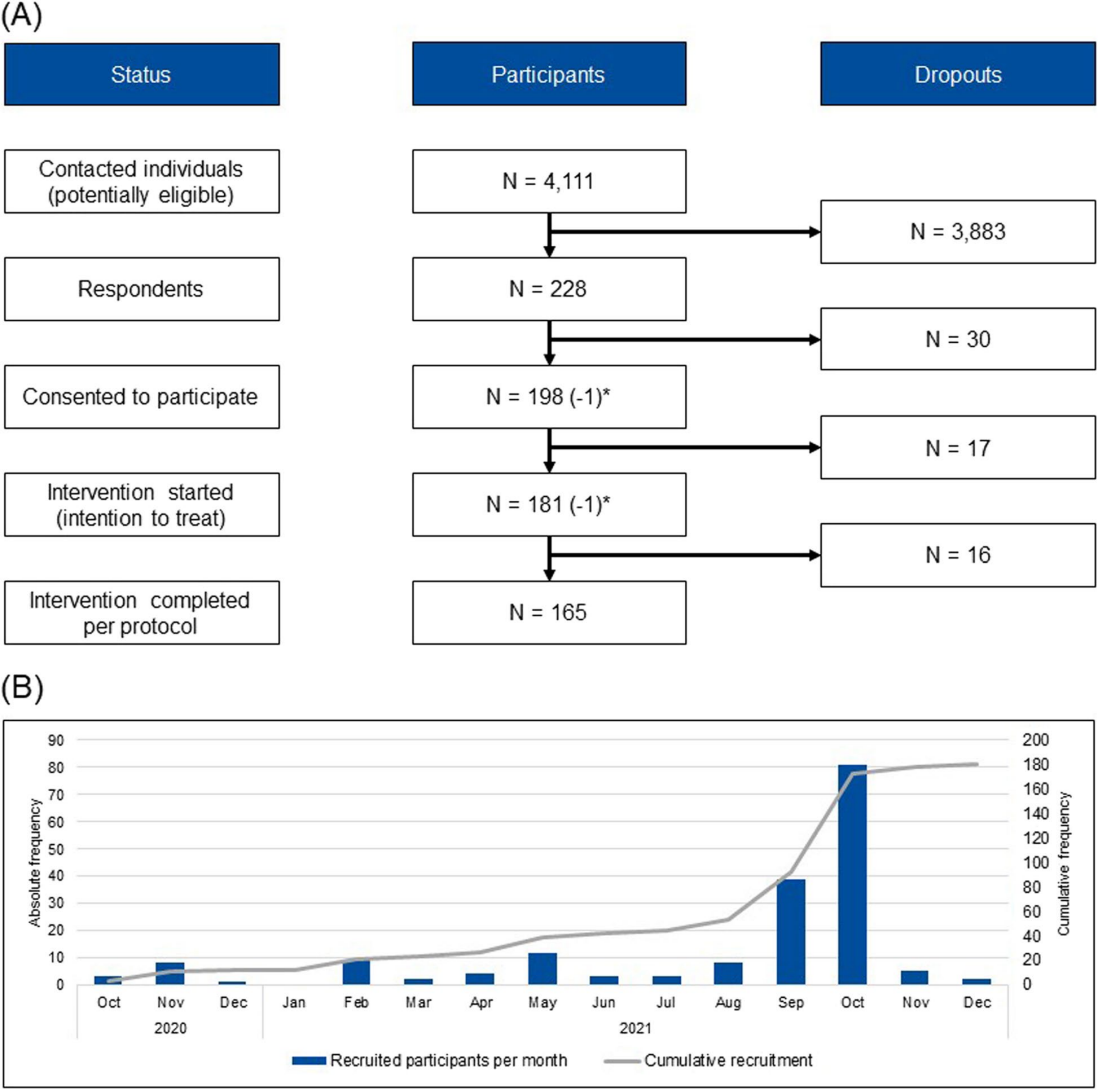
(1) Flow chart of participation and (2) recruitment schedule displaying participants recruited per month. One participant (*) started the intervention, but did not consent to the transfer of health insurance data. Their responses to the t_0_ survey were included in the analyses for the present article; they did not participate in the t_1_ survey

We received 228 responses from contacted participants (4,111 mailings). Of those, 32 respondents did not consent to participate, and 17 respondents consented initially with the health insurance companies, but did not provide consent with the social service as part of the intervention. The remaining *N* = 181 respondents participated in the intervention (ITT). Sixteen participants dropped out due to death, relocating, or other reasons leaving *n* = 165 participants to complete the 12 months of intervention (PP). Figure 1 displays the flowchart for the IG from initial mailings to final sample size after 12 months of intervention.

Based on the inclusion criteria the three health insurance companies provided *N* = 7,300 client records. Of these, *n* = 181 participants were from the IG, *n* = 17 were dropouts from the IG before the intervention started, and *n* = 7,102 were potential comparison group individuals. One individual from the IG had to be excluded due to missing data in all variables following lack of consent, and *n* = 15 individuals from the CG were excluded, because no data on the number of physician contacts in the six months prior to study initiation were provided. Overall, *n* = 7,267 individuals were matched: *n* = 180 from the IG and *n* = 7087 from the CG. Table 1 displays the socio-demographic characteristics of the entire population, the IG, and the matched CG.

**Table 1:** Socio-demographics of the IG, the matched CG, and the total CG

Variable	Label	IG	Matched CG	Total CG
**N**	-	180	708	7,087
**Sex**	female	122 (68%)	483 (68%)	4,318 (61%)
	male	58 (32%)	225 (32%)	2,769 (39%)
**Age at t**_**0**_	75–79 years	65 (36%)	261 (37%)	3,633 (51%)
	80–84 years	69 (38%)	284 (40%)	2,460 (35%)
	85 + years	46 (26%)	163 (23%)	994 (14%)
**Health insurance provider**	Insurance 1	33 (18%)	129 (18%)	1,219 (17%)
	Insurance 2	89 (49%)	351 (50%)	2,831 (40%)
	Insurance 3	58 (32%)	228 (32%)	3,037 (43%)
**Care level**	0	147 (82%)	586 (83%)	5,977 (84%)
	1	11 (6%)	35 (5%)	246 (3%)
	2	22 (12%)	87 (12%)	864 (12%)
**Number of hospitalisations before t**_**0**_	0	148 (82%)	586 (83%)	6,002 (85%)
	1	23 (13%)	94 (13%)	789 (11%)
	2	7 (4%)	20 (3%)	211 (3%)
	3	2 (1%)	6 (1%)	54 (1%)
	4	-	1 (0%)	24 (0%)
	5	-	-	4 (0%)
	6	-	1 (0%)	3 (0%)
**Number of outpatient visits**	0–5	35 (19%)	171 (24%)	2,600 (37%)
	6–10	84 (47%)	380 (54%)	3,088 (44%)
	11 +	61 (34%)	157 (22%)	1,399 (20%)

*IG* Intervention group, *CG* Control group

### Intervention use

The social service documented 104 deployments of volunteers or employees for 11 participants (range 1–37 deployments per participant), e.g., as a help for grocery shopping, physician visits, or as part of their consulting service. Telephone calls were not documented. The self-reports from participants revealed that 58/155 respondents (37%) used telephone calls with volunteers at least once. Asked about further services aside from telephone calls, six participants (4%) reported using specific services such as laundry, accompaniment, computer course, or coffee.

The social service reported 1,897 alarms from 147 participants ranging between 1 and 59 alarms per participant. Of the 1,897 alarms, 55 alarms were emergencies (3%) triggered mostly automatically (47%) and 14 alarms followed deviations within the dashboard (1%). The majority of the 55 emergencies triggered resolved without serious consequences, e.g., the contact person was informed who then reported that everything was fine with the participant (12/55), subsequent coding of “no assistance required” (11/55), or locksmith deployment that was partially cancelled (10/55). Of the remaining 22 triggered emergencies, 14 cases led to a subsequent hospitalisation, and 8 cases required assistance, but they resolved without hospitalisations. All 14 alarms following deviations within the dashboard resolved without consequences. Thus, the system produced 1,897 alarms with 22 of them being “real” emergencies (1%). Table 2 provides an overview on the type of alarm, the trigger, and the frequency of occurrence.

**Table 2:** Descriptive statistics on alarms and their triggers

Type of alarm	Total (N, %)	Trigger		
		**Manually**	**Automatically**	**Not coded**
Accidentally triggered	951 (50%)	243 (26%)	684 (72%)	24 (3%)
Notification	324 (17%)	304 (94%)	11 (3%)	9 (3%)
Test	268 (14%)	207 (77%)	54 (20%)	7 (3%)
Technical notification	220 (12%)	9 (4%)	131 (60%)	80 (36%)
Emergency	55 (3%)	20 (36%)	26 (47%)	9 (16%)
False alarm	26 (1%)	5 (19%)	21 (81%)	0
Deviation in dashboard	14 (1%)	0	1 (7%)	13 (93%)
Not coded	28 (1%)	7 (25%)	4 (14%)	17 (61%)

### Hypotheses

Our primary hypotheses addressed differences in emergency contacts and hospitalisations between the IG and the matched CG. Table 3 provides descriptive statistics for the number of emergency contacts as well as the number and duration of hospitalisations for the ITT and PP approaches. Descriptively, IG participants and their matched CG counterparts were similar on all three aspects in their respective categories and approaches.

**Table 3:** Descriptive statistics on emergency contacts and hospitalisations within the 12 months of intervention

	**Intention to treat**		**Per protocol**	
	**IG**	**Matched CG**	**IG**	**Matched CG**
N	180	708	165	650
Number of emergency contacts				
M (SD)	0.6 (1.5)	0.7 (1.8)	0.5 (1.2)	0.7 (1.8)
Median (IQR)	0 (0–1)	0 (0–1)	0 (0–1)	0 (0–1)
Min–max	0–12	0–23	0–11	0–23
Number of hospitalisations				
Mean (SD)	0.8 (1.4)	0.6 (1.2)	0.6 (1.2)	0.6 (1.1)
Median (IQR)	0 (0–1)	1 (0–1)	0 (0–1)	0 (0–1)
Min–max	0–9	0–8	0–7	0–8
Duration of hospitalisations (in days)				
M (SD)	4.1 (9.0)	4.6 (12.4)	2.9 (7.2)	4.4 (11.8)
Median (IQR)	3 (0–3)	2 (0–2)	0 (0–1)	0 (0–2)
Min–max	0–44	0–104	0–39	0–104

*IG *intervention group, *CG* Control group, *SD *Standard deviation, *IQR *interquartile range, *Min *minimum, *max *maximum

The adjusted negative binomial regressions regarding the *number of emergency contacts* did not show significant differences between the two groups in the ITT analysis (IRR = 0.89 [95%-CI: 0.62–1.28]; *p* = 0.523, primary hypothesis I, Table 4), nor in the PP analysis (IRR = 0.76 [95%-CI: 0.51–1.13]; *p* = 0.175). Additionally, older age groups (IRR_85+_ = 2.15 [95%-CI: 1.41–3.29]; *p* < 0.001) and higher care levels at t_0_ (IRR_cl1_ = 2.50 [95%-CI: 1.29–4.86]; *p* = 0.007; IRR_cl2_ = 3.00 [95%-CI: 1.89–4.75]; *p* < 0.001) were associated with a higher probability of having emergency contacts.

**Table 4:** IRR and 95%-CI of multiple mixed negative binomial regression models for the number of emergency contacts within the 12 months intervention

	**Intention to treat**			**Per protocol**		
**Predictor**	**IRR**	**95%-CI for IRR**	***p***	**IRR**	**95%-CI for IRR**	***p***
Intercept	0.27	0.20 – 0.41	< .001	0.31	0.21 – 0.44	< .001
Group: control	ref					
Group: intervention	0.89	0.62 – 1.28	.523	0.76	0.51 – 1.13	.175
Sex: female	ref					
Sex: male	0.74	0.52 – 1.06	.102	0.73	0.50 – 1.07	.103
Age: 75–79 years	ref					
Age: 80–84 years	1.38	0.95 – 2.00	.093	1.26	0.85 – 1.87	.240
Age: 85 + years	2.15	1.41 – 3.29	< .001	2.28	1.45 – 3.60	< .001
Care level: none	ref					
Care level: 1	2.50	1.29 – 4.86	.007	2.25	1.06 – 4.76	.034
Care level: 2	3.00	1.89 – 4.75	< .001	2.86	1.74 – 4.70	< .001
# of hospitalisations: 0	ref					
# of hospitalisations: 1	1.27	0.79 – 2.02	.319	1.34	0.80 – 2.25	.270
# of hospitalisations: ≥ 2	1.51	0.73 – 3.13	.271	1.54	0.65 – 3.67	.331

Observations_ITT_ = 888; N_ITT_ = 180; σ^2^_ITT_ = 1.61; ICC_ITT_ = 0.15; marginal R^2^_ITT_ = .16; conditional R^2^_ITT_ = .29; Observations_PP_ = 815; N_PP_ = 165; σ^2^_PP_ = 1.65; ICC_PP_ = 0.14; marginal R^2^_PP_ = .15; conditional R^2^_PP_ = .27; IRR = incidence rate ratio; sex, age, and care level were measured at t_0_; the number of hospitalisations was counted two quarters before t_0_ until t_0_

The IG and the matched CG did not differ in their number of hospitalisations in adjusted negative binomial regressions for ITT (IRR = 1.23 [95%-CI: 0.95–1.60]; *p* = 0.122) and PP (IRR = 0.98 [95%-CI: 0.73–1.32]; *p* = 0.905). Regarding other patient characteristics, one hospitalisation before t_0_ (IRR = 2.48 [95%-CI: 1.75–3.52]; *p* < 0.001) or two and more hospitalisations before t_0_ (IRR = 6.13 [95%-CI: 3.88–9.68]; *p* < 0.001) were associated with more hospitalisations during the intervention period in contrast to those participants who were not hospitalised before t_0_. Age, sex, and care level were not substantially associated with the number of hospitalisations. Results for the models can be found in Table S1 of the supplementary material. The IG had descriptively slightly shorter hospital stays of on average 3–4 days following linear and zero-inflated regression models in both the ITT (M_Diff_ = −2.79 [95%-CI: −7.63–2.06]; *p* = 0.260) and the PP approach (M_Diff_ = −4.33 [95%-CI: −9.43–0.76]; *p* = 0.096). However, these differences were not statistically robust. Results for the models can be found in Table S2 of the supplementary material.

### Secondary outcomes

We expected lower costs in the IG compared to the matched CG. Table 5 displays the descriptive costs for hospitalisations and overall healthcare including inpatient and outpatient care as well as transport and home care stratified by sex.

**Table 5:** Costs for hospitalisation and overall stratified by sex and group in € per person

		**Intention to treat**		**Per protocol**	
**Variable**	**Value**	**IG**	**Matched CG**	**IG**	**Matched CG**
Hospitalisations					
Total	N	180	708	165	650
	Mean (SD)	3329 (9163)	3026 (8989)	2259 (5676)	3161 (8882)
	Median (IQR)	0 (0–2952)	0 (0–2216)	0 (0–1693)	0 (0–2216)
Women	N	122	483	112	445
	Mean (SD)	3915 (10,422)	2744 (7917)	2452 (5826)	3036 (9519)
	Median (IQR)	0 (0–3373)	0 (0–1752)	0 (0–2660)	0 (0–1409)
Men	N	58	225	53	205
	Mean (SD)	2098 (5554)	3465 (10,428)	1851 (5375)	3432 (7323)
	Median (IQR)	0 (0–275)	0 (0–2636)	0 (0–101)	0 (0–3040)
Overall costs					
Total	N	180	708	165	650
	Mean (SD)	3691 (9398)	3766 (10,354)	2599 (5952)	3751 (9863)
	Median (IQR)	34 (0–2952)	0 (0–2217)	0 (0–1693)	0 (0–2216)
Women	N	122	483	112	445
	Mean (SD)	4186 (10,622)	3569 (9553)	2685 (6008)	3739 (10,697)
	Median (IQR)	19 (0–3373)	0 (0–1752)	0 (0–2660)	0 (0–1409)
Men	N	58	225	53	205
	Mean (SD)	2651 (6019)	4074 (11,487)	2418 (5886)	3778 (7774)
	Median (IQR)	105 (0–275)	0 (0–2636)	63 (0–101)	0 (0–3040)

*IG* Intervention group, *CG *Control group, *SD* Standard deviation, *IQR* Interquartile range displayed as 25. percentile – 75. percentile

According to our multiple regression analyses for overall costs, the probability of producing costs was similar in both groups (OR in ITT: 0.86, 95%-CI: 0.60–1.22, OR in PP: 1.01, 95%-CI: 0.70–1.46). Costs for those with any costs were similar and only slightly less for the IG compared to the CG (on average in ITT: −7%, 95%-CI: −54%−40%, in PP: −32%, 95%-CI: −82%−19%). In contrast, one or two and more hospitalisations before t_0_, second care level at t_0_, and age between 80 and 84 years compared to younger aged patients were strongly associated to higher overall costs. None of the other variables were substantially associated to costs. The multiple regression analyses for overall costs can be found in Table S3 of the supplementary material.

Lastly, we expected that the IG participants had better health outcomes and fall related perceptions at the end of the intervention (t_1_) compared to the beginning (t_0_). Regarding self-reported falls, *n* = 172 participants responded at t_0_, and *n* = 163 participants at t_1_. For t_0_, 61/172 participants (35%) reported having fallen in the previous 12 months compared with 61/163 participants (37%) during the intervention at t_1_. The number of falls did not differ substantially between t_0_ (*M* = 1.95; *SD* = 1.63) and t_1_ (*M* = 1.91; *SD* = 2.24) (*M*_*diff*_ = 0.04 [95%-CI: −0.69–0.77]; *t*(109) = 0.11; *p* = 0.91). Table 6 shows descriptive statistics and t-test results for the survey data regarding the constructs concerns about falling, loneliness, social support, HR-QOL physical and mental scores, perceived stress, (I)ADL, and TC. Participants reported mainly negative outcomes at t_1_ compared to t_0_, namely higher concerns about falling and perceived stress, as well as lower social support, HR-QOL physical health, and (I)ADL.

**Table 6:** Descriptive results and comparisons of self-reports in the IG between t_0_ and t_1_ following intention to treat

Variable	N	M (SD) t_0_	M (SD) t_1_	M Diff (t_1_-t_0_) [95%-CI]	*t*	*p*	*d*
Concerns about falling	159	1.6 (0.6)	1.8 (0.7)	0.2 [0.1–0.3]	5.85	< .001	0.46
Social support	156	4.0 (0.7)	3.8 (0.7)	−0.2 [−0.3–0.1]	−3.42	< .001	−0.60
HR-QOL, physical score	126	40.4 (8.7)	37.6 (8.0)	−2.8 [−4.1–1.4]	−4.15	< .001	−0.37
HR-QOL, mental score	126	42.1 (5.7)	42.2 (5.9)	0.1 [−1.1–1.2]	0.14	.892	-
Perceived stress, full scale	157	24.2 (6.9)	25.8 (7.5)	1.6 [0.6–2.7]	3.07	.003	0.25
Perceived stress, helplessness	157	14.2 (4.8)	15.7 (5.1)	1.5 [0.7–2.2]	3.79	< .001	0.30
Perceived stress, self-efficacy	157	13.3 (3.4)	13.0 (3.3)	−0.3 [−0.9–0.3]	−1.14	.255	-
ADL	160	97.2 (6.4)	94.3 (10.4)	−2.8 [−4.2–1.5]	−4.23	< .001	−0.34
IADL	159	7.5 (1.1)	7.2 (1.5)	−0.3 [−0.5–0.2]	−3.65	< .001	−0.29
TC Competence	169	3.2 (1.2)	-	-	-	-	-
TC Control	168	3.4 (0.9)	-	-	-	-	-
TC Acceptance	169	2.6 (1.0)	-	-	-	-	-

*M* Mean, *SD* Standard deviation, *M*_diff_ Mean difference between t_0_ and t_1_, *HR-QOL*Health-related quality of life, *ADL* Activities of daily living, *IADL i*nstrumental activities of daily living, *TC* technology commitment (only measured at t_0_)

## Discussion

The purpose of this study was to evaluate an integrated system for emergency prevention and support consisting of motion sensors, a home emergency call system, and a social service. We targeted community-dwelling ≥ 75-year-olds in and around Berlin, Germany, who received the integrated system and access to the social service for 12 months. The aim of our study was to assess whether the intervention could reduce contacts to the emergency departments, hospitalisation rates, and the healthcare costs compared to a matched CG. Our results revealed no significant differences between the IG and matched CG regarding the number of emergency contacts in the ITT approach, nor in the PP analysis. Regarding hospitalisations, the IG and matched CG did not differ in the number of hospitalisations. We did not find differences in the associated healthcare costs. Participants of the IG reported higher perceived stress and concerns about falling as well as lower HR-QOL and social support at the end of the 12-months intervention period compared to the beginning. However, as these data were only available for the IG and not for the CG, the data are not indicative of any effectiveness of the intervention.

Previous intervention studies reported similar rates [16] or more hospitalisations in the IG [14, 15]. For example, although Schindel et al. [14] reported effectiveness of their intervention regarding decreased healthcare cost, the number of hospitalisation rates increased in both groups: in the IG, IRR = 0.37 before and IRR = 0.56 after intervention compared to CG, IRR = 0.38 before and IRR = 0.42 after intervention. More hospitalisations in the IG have been attributed to participants’ increased attention and monitoring of health in intervention studies generally, particularly for older adults [15]. Such an interpretation would also explain shorter hospital stays in general: increased attention to symptoms could lead them to seek medical care *earlier* with potentially beneficial effects for the progression of symptoms. Thus, positive effects of the intervention might be visible through shorter stays with similar rates of hospitalisations. However, we did not find robust differences between our IG and their matched controls. Thus, our intervention could not make use of the potentially protective effect of early medical attention – at least not within the 12 months intervention period. The social service in our intervention provided advice for utilising additional medical and nursing services potentially increasing healthcare costs in the short term whilst potentially addressing medical needs of this population more adequately. However, most participants of the IG reported not using the social service. Different observation periods or an active control instead of matched controls might provide additional insights into effects of interventions. Emergency contacts were significantly lower in the IG only in the PP approach, i.e., in those participants adherent to the entire duration of the intervention and with easier access to emergency departments through their home emergency call system. This finding may be attributable to a selection bias, as healthier participants were more likely to remain in the study, leading to the premature dropout of those who were severely ill or in the process of dying.

However, even within those adherent participants, perceived stress and concerns about falling were *higher* while their physical health, social support, and the (I)ADL were *lower* between the start and the end of the intervention. However, the minimal clinically important difference (MCID) for HR-QOL has been reported at ≥ 3.29 for the physical score and ≥ 3.77 for the mental score [45], which are above the difference observed in our study. For concerns about falling, the MCID was reported at ≥ 0.34 or ≥ 0.63 depending on the calculation method [46], which are above the difference observed in our study. Thus, the deterioration in some health values and the physical HR-QOL might not be relevant for participants and can presumably be attributed to the participants’ high mean age at t_0_ (*M* = 81.70 years). Indeed, it is expected that the health of very old people deteriorates over the course of a year particularly if it is below the MCID. The survey results should be interpreted accordingly, particularly since they are lacking data from their matched counterparts who have not been surveyed as part of this study design. Future research could employ new methods that measure outcomes repeatedly, including methods such as ecological momentary assessments, throughout the entire intervention period to assess its short-term effects. While various explanations for reduced HR-QOL in research exist, including natural declines over time or other life circumstances, it is also plausible that imposing a lifestyle change due to an intervention adversely affects HR-QOL [47]. Participants did not report higher incidence of falling between the 12 months prior to the intervention and the intervention period supporting the decline below MCID as not detectable by participants. With 35% and 37% of participants reporting falls, respectively, our sample closely mirrors other studies on falls in older adults, which report a falling incidence of 36% [48].

The evaluated intervention is part of the family of telemedical interventions or AAL systems. These interventions promise immediate responses particularly relevant for people living alone who would otherwise not receive help quickly. AAL systems can delay or prevent the need to move to a care home, and they focus on physical functioning, (I)ADL, cognitive functioning, or social skills [49]. A scoping review of 54 studies found promising effects of AAL systems particularly in the context of dementia care [50]. A recent systematic mapping review identified 233 studies with AAL technologies predominantly from USA, UK, Japan, or continental Europe that focus mainly on ADL, fall detection, or activity and movement monitoring [51]. However, unlike the scoping review, many of those studies only introduced the technologies, but did not investigate their effectiveness, or they disregarded clinical aspects and focused on cost-effectiveness [51]. Our present matched-control study is best compared to the Bieg et al. [23] study regarding the sample size and mixed-methods design with similar findings regarding self-reports between t_0_ and t_1_. If the effectiveness of AAL systems had generally been demonstrated, particularly rural areas with low density of medical professionals per inhabitants could benefit since those systems do not rely on physical proximity. In the case of our intervention, we exclusively targeted inhabitants of urban outskirts. Thus, our results are not indicative for rural areas.

The effectiveness of interventions with technological components depends among other factors on the ‘technological readiness’ or ‘digital literacy’ of the users [42]. Yet older adults regularly exhibit lower rates of technological uptake and digital literacy than younger groups [52]. Our survey results revealed comparably low TC as measure for readiness or literacy (2.6 ≤ *M* ≤ 3.2 for the three sub-scales). Other studies report somewhat higher TC ratings (3.2 ≤ *M* ≤ 4.1) for a similar target group [53, 54]. Lower TC in our sample potentially means that participants did not interact with novel technologies as intuitively and competently as others with higher TC. However, a systematic review on 83 studies highlighted the role of varying personal factors, the social context, and the design of technological elements for successful use of technologies of older adults [55]. The system used in the present study operated mainly in the background (sensors), and its interactive components were designed in a way to make them easy to use, i.e., with large emergency buttons on the pendant and the base station. We thus consider technological readiness not as paramount for successful implementation compared to other more interactive digital technologies such as smartphone apps.

As many other studies conducted in recent years, our study suffered from the restrictions imposed by the COVID-19 pandemic restrictions [56]. We originally planned to survey IG participants in person in their households and to include hand strength measures as proxy for physical fitness. Such an in-person visit would provide additional insights into the circumstances and fitness of the participants. Due to contact restrictions, we surveyed participants via telephone or sent the survey via mail, and we suspended the hand strength measures. The severe restrictions and burdens especially for the COVID-19 high-risk group of ≥ 75-year-olds may also partly explain the survey results, i.e., in the form of poorer HR-QOL or reduced social support. Again, survey results were only recorded for participants of the IG, not for their matched controls. Thus, we cannot assess differences between groups. Recent empirical data (*N* = 48,356 older participants) indicate that self-rated health deteriorated during the course of the pandemic, and health disparities widened among older adults in Europe [57]. This general observation of the overall negative health impact of the pandemic must be considered when interpreting the present results.

### Strengths and limitations

The tested intervention offered a novel technological system that was easy to use together with access to a social service provider that offered personalised services according to individual needs and wishes. Methodologically, we used a matched-control study that provides a trustworthy level of evidence particularly compared to observation studies, user feedback, or heuristic expert evaluations. The sample size of 181 IG participants and 708 matched controls outnumbers many AAL studies, which can be seen as a clear strength of this study. Additionally, 165 participants completed the 12-months intervention period resulting in a 9% dropout rate, which is comparably low for intervention studies in this field. Furthermore, our study linked survey data with insurance claims data. This mix of different data sources provides insights into the real world and subjective health data of community-dwelling older adults. We were thus able to link hospitalisation rates with self-reports from participants, which is a strength of this study.

A few limitations must be outlined. Self-reports and health insurance claims data provide valuable information, albeit with limitations. Self-reports are inherently subjective and prone to recall bias, particularly regarding the number of falls in the previous 12 months. Health insurance claims data only include health service expenditures submitted to the insurance company. Any privately made expenditures or unpaid health services are excluded from the data. However, within these limitations our study provides valuable information regarding the health outcomes from a complex intervention. Further, the findings may be influenced by selection bias, as participants who were healthier tended to remain in the study, while those who were severely ill or nearing death dropped out earlier. This attrition could distort the results and affect the overall validity of the conclusions drawn from the data. Another limitation of the present study includes the technical components. The technical system as tested in our study is no longer available from the provider as the entire department has been restructured and the sensor- and necklace-based system is no longer offered commercially. This limitation exemplifies a general challenge for technology-based health interventions: technological advancements occur rapidly, often outpacing the scientific implementation and evaluation. This phenomenon has been previously described as technological obsolescence [58]. Technological obsolescence occurs when older technologies become less useful due to the availability of newer, superior alternatives, or when support for the older versions ceases. This decline reduces their initial value and potentially limits their future functionality [58]. Given the constraints on resources, research and health organisations are increasingly recognising the importance of revaluating potentially ineffective, wasteful, and outdated health technologies. This reassessment could allow for the reallocation of funds to more clinically effective technologies, thereby enhancing the quality of care and improving public health outcomes [59]. Based on the results and the experiences made within the present study, we strongly recommend that future research involving new technologies allocate sufficient resources to keep pace with technological advancements. This will ensure the implementation and evaluation of technology-based approaches that remain current and effective. Lastly, we did not collect data on cognitive ability of the participants, which can influence the fall rates.

### Conclusion

The present study evaluated a complex intervention consisting of motion sensors, a home emergency call system, and a social service for community-dwelling older people using a matched-control study design. The intervention had no effect on hospitalisations and emergency contacts in our IG. Thus, the low-threshold offer did not materially change the participants’ health outcomes. These findings present an opportunity to reflect on the design of AAL interventions. Further technological advances are needed for similar interventions to achieve better outcomes; alternatively, other types of systems could be tested for their efficacy.

## Supplementary Information

Supplementary Material 1

Supplementary Material 2

## Data Availability

The datasets used and/or analysed during the current study are available from the corresponding author on reasonable request and with the permission of the insurance companies for claims data.
