# Supplementary material for: Fall sensors, home emergency system, and social service for ≥ 75-year-olds living at home - a matched control intervention study
Source: BMC Geriatr. 2025 Apr 2;25:217. doi: 10.1186/s12877-025-05856-2 (PMC11963553; doi:10.1186/s12877-025-05856-2)
Supplement: Supplementary file 1 — Supplementary Material 1 [file 12877_2025_5856_MOESM1_ESM.docx]

Table S1

*IRR and 95%-CI of multiple mixed negative binomial regression models for the number of hospitalisations within the 12 months intervention*

|  | **Intention to treat** | | | **Per protocol** | | |
| --- | --- | --- | --- | --- | --- | --- |
| **Predictor** | **IRR** | **95%-CI for IRR** | ***p*** | **IRR** | **95%-CI for IRR** | ***p*** |
| Intercept | 0.32 | 0.24 – 0.42 | <.001 | 0.32 | 0.24 – 0.43 | <.001 |
| Group: control | Ref |  |  |  |  |  |
| Group: intervention | 1.23 | 0.95 – 1.60 | .122 | 0.98 | 0.73 – 1.32 | .905 |
| Sex: female | ref |  |  |  |  |  |
| Sex: male | 1.01 | 0.76 – 1.34 | .962 | 1.00 | 0.74 – 1.37 | .988 |
| Age: 75-79 years | ref |  |  |  |  |  |
| Age: 80-84 years | 1.23 | 0.92 – 1.65 | .161 | 1.28 | 0.94 – 1.75 | .119 |
| Age: 85+ years | 1.05 | 0.74 – 1.50 | .774 | 1.05 | 0.71 – 1.55 | .818 |
| Care level: none | ref |  |  |  |  |  |
| Care level: 1 | 1.49 | 0.86 – 2.59 | .159 | 1.36 | 0.71 – 2.62 | .359 |
| Care level: 2 | 1.41 | 0.95 – 2.08 | .088 | 1.31 | 0.85 – 2.03 | .223 |
| # of hospitalisations: 0 | ref |  |  |  |  |  |
| # of hospitalisations: 1 | 2.48 | 1.75 – 3.52 | <.001 | 2.42 | 1.63 – 3.58 | <.001 |
| # of hospitalisations: ≥2 | 6.13 | 3.88 – 9.68 | <.001 | 6.76 | 3.97 – 11.48 | <.001 |

*Note*. Observations_ITT_ = 888; N_ITT_=180; σ²_ITT_ = 1.24; ICC_ITT_ = 0.16; marginal R²_ITT_ = .15; conditional R²_ITT_ = .29; Observations_PP_ = 815; N_PP_ = 165; σ²_PP_ = 1.29; ICC_PP_ = 0.17; marginal R²_PP_ = .12; conditional R²_PP_ = .27; IRR = incidence rate ratio; sex, age, and care level were measured at t_0_; the number of hospitalisations was counted two quarters before t_0_ until t_0_.

Table S2

*Estimates and 95%-CI of linear and zero-inflated regression models for the duration of hospitalisations within the 12 months intervention*

|  | **Intention to treat** | | | **Per protocol** | | |
| --- | --- | --- | --- | --- | --- | --- |
| **Predictor** | **M_Diff_** | **95%-CI for M_Diff_** | ***p*** | **M_Diff_** | **95%-CI for M_Diff_** | ***p*** |
| **Linear model** |  |  |  |  |  |  |
| Intercept | 12.73 | 8.48 – 16.97 | <.001 | 12.65 | 8.10 – 17.21 | <.001 |
| Group: control | ref |  |  |  |  |  |
| Group: intervention | -2.79 | -7.63 – 2.06 | .260 | -4.33 | -9.43 – 0.76 | .096 |
| Sex: female | ref |  |  |  |  |  |
| Sex: male | -3.01 | -7.16 – 1.14 | .155 | -3.30 | -8.05 – 1.45 | .174 |
| Age: 75-79 years | ref |  |  |  |  |  |
| Age: 80-84 years | 3.11 | -1.53 – 7.75 | .189 | 2.45 | -2.57 – 7.48 | .338 |
| Age: 85+ years | 2.09 | -3.23 – 7.40 | .442 | 1.85 | -4.21 – 7.91 | .550 |
| Care level: none | ref |  |  |  |  |  |
| Care level: 1 | -2.93 | -10.02 – 4.16 | .419 | -1.99 | -11.35 – 7.38 | .678 |
| Care level: 2 | 3.41 | -1.91 – 8.73 | .209 | 2.71 | -3.66 – 9.08 | .405 |
| # of hospitalisations: 0 | ref |  |  |  |  |  |
| # of hospitalisations: 1 | -1.43 | -6.16 – 3.29 | .552 | 0.59 | -5.25 – 6.43 | .843 |
| # of hospitalisations: ≥2 | 19.85 | 13.34 - 26.36 | <.001 | 16.02 | 8.55 – 23.50 | <.001 |
|  |  |  |  |  |  |  |
| **Zero-inflated model** | **OR** | **95%-CI for OR** | ***p*** | **OR** | **95%-CI for OR** | ***p*** |
| Group: control | ref |  |  |  |  |  |
| Group: intervention | 0.96 | 0.66 - 1.38 | .810 | 1.17 | 0.79 - 1.73 | .441 |
| Sex: female | ref |  |  |  |  |  |
| Sex: male | 0.78 | 0.57 - 1.06 | .118 | 0.73 | 0.53 - 1.02 | .066 |
| Age: 75-79 years | ref |  |  |  |  |  |
| Age: 80-84 years | 0.93 | 0.66 - 1.32 | .703 | 0.92 | 0.64 - 1.31 | .645 |
| Age: 85+ years | 0.94 | 0.63 - 1.4 | .769 | 0.9 | 0.59 - 1.39 | .657 |
| Care level: none | ref |  |  |  |  |  |
| Care level: 1 | 0.3 | 0.16 - 0.56 | <.001 | 0.47 | 0.23 - 0.95 | .035 |
| Care level: 2 | 0.43 | 0.28 - 0.66 | <.001 | 0.53 | 0.33 - 0.84 | .007 |

*Note*. Observations_ITT_ = 888; N_ITT_=180; σ²_ITT_ = 16.28; ICC_ITT_ = 0.00; marginal R²_ITT_ = .58; conditional R²_ITT_ = .58; Observations_PP_ = 815; N_PP_ = 165; σ²_PP_ = 14.51; ICC_PP_ = 0.73; marginal R²_PP_ = .22; conditional R²_PP_ = .79; M_Diff_ = Mean difference; sex, age, and care level were measured at t_0_; the number of hospitalisations was counted two quarters before t_0_ until t_0_. Log OR = logarithm odds ratios

Table S3

*Estimates and 95%CI of zero inflated linear mixed regression models for the overall healthcare costs within the 12 months intervention*

|  | **Intention to treat** | | | **Per protocol** | | |
| --- | --- | --- | --- | --- | --- | --- |
| **Predictor** | **β** | **95%-CI for β** | ***p*** | **β** | **95%-CI for β** | ***p*** |
| **Linear model** |  |  |  |  |  |  |
| Intercept | 6.84 | 6.43 – 7.24 | <.001 | 6.82 | 6.41 – 7.24 | <.001 |
| Group: control | ref |  |  |  |  |  |
| Group: intervention | -0.07 | -0.54 – 0.40 | .774 | -0.32 | -0.82 – 0.19 | .216 |
| Sex: female | ref |  |  |  |  |  |
| Sex: male | 0.32 | -0.10 – 0.74 | .136 | 0.47 | 0.03 – 0.91 | .037 |
| Age: 75-79 years | ref |  |  |  |  |  |
| Age: 80-84 years | 0.58 | 0.12 – 1.05 | .015 | 0.62 | 0.14 – 1.10 | .011 |
| Age: 85+ years | 0.14 | -0.38 – 0.66 | .606 | 0.13 | -0.42 – 0.68 | .643 |
| Care level: none | ref |  |  |  |  |  |
| Care level: 1 | 0.63 | -0.10 – 1.36 | .092 | 0.48 | -0.35 – 1.31 | .253 |
| Care level: 2 | 0.55 | 0.03 – 1.07 | .038 | 0.50 | -0.05 – 1.05 | .076 |
| # of hospitalisations: 0 | ref |  |  |  |  |  |
| # of hospitalisations: 1 | 0.49 | 0.00 – 0.98 | .048 | 0.50 | -0.03 – 1.03 | .062 |
| # of hospitalisations: ≥2 | 1.67 | 0.93 – 2.42 | <.001 | 1.93 | 1.08 – 2.78 | <.001 |
|  |  |  |  |  |  |  |
| **Zero-inflated model** | **Log OR** | **95%-CI for log OR** | ***p*** | **Log OR** | **95%-CI for log OR** | *p* |
| Group: control | ref |  |  |  |  |  |
| Group: intervention | 0.86 | 0.6 - 1.22 | .395 | 1.01 | 0.7 - 1.46 | .959 |
| Sex: female | ref |  |  |  |  |  |
| Sex: male | 1.13 | 0.83 - 1.52 | .459 | 1.13 | 0.82 - 1.55 | .462 |
| Age: 75-79 years | ref |  |  |  |  |  |
| Age: 80-84 years | 0.9 | 0.65 - 1.25 | .526 | 0.9 | 0.65 - 1.27 | .575 |
| Age: 85+ years | 0.66 | 0.45 - 0.98 | .040 | 0.68 | 0.45 - 1.02 | .064 |
| Care level: none | ref |  |  |  |  |  |
| Care level: 1 | 0.3 | 0.14 - 0.63 | .001 | 0.32 | 0.15 - 0.68 | .004 |
| Care level: 2 | 0.29 | 0.18 - 0.47 | <.001 | 0.31 | 0.19 - 0.51 | <.001 |
| # of hospitalisations: 0 | ref |  |  |  |  |  |
| # of hospitalisations: 1 | 0.22 | 0.14 - 0.36 | <.001 | 0.25 | 0.15 - 0.41 | <.001 |
| # of hospitalisations: ≥2 | 0.09 | 0.03 - 0.27 | <.001 | 0.08 | 0.02 - 0.29 | <.001 |

*Note*. Observations_ITT_ = 888; N_ITT_=180; σ²_ITT_ = 2.05; ICC_ITT_ = 0.00; marginal R²_ITT_ = .12; conditional R²_ITT_ = .12; Observations_PP_ = 815; N_PP_ = 165; σ²_PP_ = 2.01; ICC_PP_ = 0.00; marginal R²_PP_ = .13; conditional R²_PP_ = .13; sex, age, and care level were measured at t_0_; the number of hospitalisations was counted two quarters before t_0_ until t_0_.
