# Supplementary material for: Fall sensors, home emergency system, and social service for ≥ 75-year-olds living at home - a matched control intervention study
Source: BMC Geriatr. 2025 Apr 2;25:217. doi: 10.1186/s12877-025-05856-2 (PMC11963553; doi:10.1186/s12877-025-05856-2)
Supplement: Supplementary file 2 — Supplementary Material 2 [file 12877_2025_5856_MOESM2_ESM.docx]

**S1. Guideline for interviews with participants**

| ***Topic and Concept*** |
| --- |
| ***Opening Question*** |
| ***Please start by telling me about your professional journey to SOPHIA*** |
| ***A work routine*** |
| ***1.1 Work routineTo help me better understand your work, could you describe what your last workday was like? Please tell me about it***  *Possible follow-up questions:*  *What happens in detail?*  *Which experience stands out the most in your memory?*  *Are there any other experiences?*  *What obstacles do you perceive?*  *How do you experience that?*  ***1.2 Was that a typical day? If not: How would you describe a typical day?***  ***2.Differences***  ***2.1 In what ways does working on the VBW project differ from your usual work routine at SOPHIA?***  *Possible follow-up questions:*  *Could you describe the advantages and disadvantages of working on the VBW project?*  *Can you provide examples?* |
| ***B Technology and Care*** |
| ***1.*** ***Use and Experience of Technology (Personal)***  *As part of the VBW project, a home emergency call system with fall detectors and motion sensors was installed with the goal of enabling a longer and safer stay in one's own home. The following questions specifically relate to this technology integrated within the project.*  ***1.1 How do you experience working with the technology installed by Philips in the project? Please comment on the different devices, if possible.***   - 1. ***How has working with the technology changed over the course of the project?***   *Possible follow-up questions:*  *Can you provide specific examples or situations?*  *What advantages and disadvantages can you report?*  *What happens in detail?*  *How do you experience this?*  *How do you handle it?*  ***2. Technology Use/Experience of Participants***  ***2.1 How do the participants handle the technology?***  ***2.2 What do the participants report to you***  *Possible Follow-up Question:*  *What specific benefits and challenges do they report?*  ***3.*** ***Care and Support for Participants***  ***3.1 How do the participants perceive the services and offerings?***  *Possible Follow-up Question:*  *What feedback or reactions do you receive?* |

| ***C Collaboration within the Consortium*** |
| --- |
| ***1.Challenges in the Project Consortium***   - 1. ***How do you experience working on the project with the different partners? Please share your thoughts.***   2. ***What works well, and what doesn't?***   ***1.3 Can you elaborate on that, please?***  *Possible Follow-up Questions:*  *How would you describe the collaboration?*  *What challenges do you encounter? Please tell me more about the conflicts you mentioned.*  *How do you handle these situations?*  *Can you provide specific examples?* |
| ***D Future SOPHIA*** |
| ***1.Future***  ***1.1 When you look 2-3 years into the future, how do you envision the use of technology at SOPHIA?***  ***1.2 Tell me about the wishes you have for SOPHIA's work.***  *Possible follow-up questions:*  *Can you imagine that the project technology will continue to be integrated into SOPHIA's daily operations?*  *2a) If yes - In what way or under what circumstances?*  *2b) If no - Why not?* |
| ***E Closing*** |
| ***If you reflect on our conversation, is there anything else you would like to mention or emphasize? Please take your time.*** |

**S2. Guideline for focus group with participants**

Guiding Questions:

How do you currently assess the new care model/project (as a suggestion)?

What did you hope to gain from participating in the study before it started?

How did you experience the entire study participation?

What benefits do you see in the new care model/project?

What did you like? What didn’t you like? How would an optimal outcome look?

Procedure:

Welcome/Refreshments

Introduction Round including the announcement: Thank you very much for coming and for your willingness to share your experiences in virtual assisted living. We look forward to your constructive and critical contributions in order to evaluate the project in the final stage.

*Short promotional video about VBW as an introduction and to start the topic*

**1st Discussion Round – Reasons for Participation:**

These are the reasons for participation provided by participants in the Virtual Assisted Living. Can you go through these together? How do you see it? There is no right or wrong here. We are interested in your perspective.

- (free) offer from the health insurance company ‘I used the service because it was offered by my health insurance company.’
- living alone ‘I live alone and the program means that someone is always there for me.’
- being alone ‘Thanks to the program, I no longer feel so alone.’
- Age ‘At my age, it makes sense to take advantage of such an offer.’
- Relieving the burden on relatives ‘The service relieves the burden on my relatives.’
- Relatives' request/recommendation ’The service was recommended to me by relatives.’
- Trying out/preventive benefit ‘The program enables me to prevent injuries.’
- Health restrictions/problems ‘Due to my health restrictions, I need help, which the program can give me.’
- Help when needed ‘Thanks to the program, I get help when I need it.’
- Help in emergencies ‘The program provides me with support in emergencies.’
- Interest/ occupation ‘I am interested in the offer and would like to deal with the topic.’
- No/little support/support/help ‘At the moment I receive little/no support and the program would mean that someone would be there for me.’
- Self-determination ‘I would like to lead a self-determined life.’
- Maintaining independence ‘I would like to manage my everyday life independently.’
- Need for security ‘I would like security in my own home.’
- Concerns/fear of falling/risk of falling ‘I am worried about falling in my home.’
- Supporting a scientific research project ‘I would like to support research by participating in the study.’
- Desire to remain in my home ‘The offer enables me to continue living in my home.’

Can you discuss and weigh the importance of these reasons together, and rank them in a hierarchical pyramid? The higher a reason is placed, the more important it is.

**2nd Discussion Round – Positives/Negatives/Improvements:**

Guiding Questions: What did you like? What didn’t you like? How would an optimal outcome look like?

*Participants should discuss the advantages and disadvantages, and ideally design a scenario (as a collective strategy for problem-solving) “Suggestions for improving the intervention/study process.”*

Now, focus on your positive and negative experiences with Virtual Assisted Living. The (index cards) contain positive and negative comments that have already been mentioned. They are just a starting point or a reminder. Please exchange your experiences and discuss how things could have been better or optimal.

**S3. Guideline for focus group with project partners**

Two main topics:

Evaluation of the form of care and evaluation of the project implementation

Procedure:

Welcome/ hospitality

Round of introductions

Introduction via project flyer: In front of you is the flyer that we created about the project at the beginning of VBW. There you can see the project goals and orientation. If you compare your experience in the project with the flyer, what are your impressions? What has come true? What has changed? How do you see the Virtual Assisted Living project today compared to the approach three years ago?

**1st discussion round – Evaluation of the implementation of the project**

Guiding Questions:

How do you rate the implementation of the project?

- What worked well?
- Where did obstacles arise?
- What could have gone better?
- How has the COVID-19 pandemic affected the project? Where could we have reacted better and how?

What should future projects consider?

- What lessons have you learned regarding the implementation of such a project?
- What should be considered during planning?
- What should be done differently (e.g. resources, recruitment planning, composition of the consortium)?

**2nd discussion round – Evaluation of the form of care:**

- What is your current assessment of the new form of care?
- In your opinion, what are the benefits of the new form of care?
- What risk factors/barriers does the new form of care have?
- What do you think the future holds for the new form of virtual assisted living? (Transfer to standard care? Follow-up projects?)

**S4. Acceptability survey for potentially eligible people**

**1. Technology commitment**

*Technology commitment (Neyer et al., 2012)*

***2. More and more technical devices are also being used today in the area of health maintenance and prevention, such as pedometers, sensors for fall detection or assistance systems that switch off the stove, for example. Such applications are often already networked. In the following questions, we are interested in your views on such technologies. The term “digital health technologies” refers to networked devices that can record and forward information about a person's state of health.***

Are you familiar with digital health technologies (e.g. from the media)? □ yes □ no

Do you already use digital health technologies yourself? □ yes □ no

|  | **Agree not at all** | **Rather not** | **Equally yes and no** | | **Rather yes** | **Completely agree** |
| --- | --- | --- | --- | --- | --- | --- |
| I find digital health technologies useful in my daily life. | □ | □ | □ | | □ | □ |
| By using digital health technologies in the home, I can better maintain my independence. | □ | □ | □ | | □ | □ |
| The use of digital health technologies in the home makes me feel monitored. | □ | □ | □ | | □ | □ |
| I can prevent health risks by using digital health technologies in the home. | □ | □ | □ | | □ | □ |
| The use of digital health technologies in the home is determined by economic interests. | □ | □ | □ | | □ | □ |
| The use of digital health technologies in the home relieves the burden on my relatives. | □ | □ | □ | | □ | □ |
| By using digital health technologies in the home, I can live in my own four walls for longer. | □ | □ | □ | | □ | □ |
| The use of digital health technologies in the home makes me feel safer. | □ | □ | □ | | □ | □ |
| The use of digital health technologies only makes my life more complicated. | □ | □ | □ | | □ | □ |
| People who are important to me think I should use digital health technologies. | □ | □ | □ | | □ | □ |
| Digital health technologies disturb me in my home. | □ | □ | □ | | □ | □ |
| Digital health technologies increase my living comfort. | □ | □ | □ | | □ | □ |
| With digital health technologies, I'm worried that information and data about me could fall into the wrong hands. | □ | □ | □ | | □ | □ |
| I fear having less contact with my family/friends due to digital health technologies. | □ | □ | □ | | □ | □ |
|  | **Not at all** | **Rather not** | **Equally yes and no** | **Rather yes** | | **Completely** |
| I like it when important information about my condition is passed on to a home emergency call system using digital technologies. | □ | □ | □ | | □ | □ |
| The connection to a home emergency call system is superfluous for me. | □ | □ | □ | □ | | □ |
| The connection to a home emergency call system gives me the feeling that I'm not alone in an emergency. | □ | □ | □ | □ | | □ |

***3. We would now like to find out more about your current health and about you as a person.***

How would you describe your state of health in general?

□ excellent □ very good □ good □ fair □ poor

Compared to one year ago, how would you rate your health in general now?

□ Much better now than one year ago □ Somewhat better now than one year ago

□ About the same □ Somewhat worse now than one year ago

□ Much worse now than one year ago

Who will support you if you need help in everyday life?

□ Relatives □ Friends/acquaintances □ Neighbours □ Household help □ No one

□ I do not need any help

Do you have a care level?

□ 1 □ 2 □ 3 □ 4 □ 5 □ no care level □ I don’t know

***4. Personal information***

Gender: □ female □ male □ diverse

Age: ______ years

What is your highest general school-leaving qualification?

□ Finished school without a school-leaving certificate
□ Secondary or elementary school certificate
□ Secondary school leaving certificate/intermediate school leaving certificate/technical school leaving certificate
□ POS (polytechnic secondary school) or 10th grade (before 1965: 8th grade)
□ Technical college entrance qualification/graduation from a technical college
□ Abitur/general or subject-specific higher education entrance qualification
□ Other school-leaving qualification (e.g. obtained abroad)

What is your highest vocational qualification?

□ No vocational qualification
□ Apprenticeship (vocational/in-company training)
□ Training at vocational school/trade school (vocational-school training)
□ Technical school (master craftsman, technical school, vocational or technical academy)
□ University of applied sciences/engineering school
□ University or college
□ Other: ___________________________________________

How do you currently live? (multiple answers possible)

□ alone □ with partner □ with relatives

□ Shared appartment □ differently: ____________________________________________

Do you intend to take part in the “Virtual Assisted Living” project?

□ yes □ no □ undecided

If you do not wish to participate, that is of course perfectly fine. Please give a brief reason why. This will help us a lot in evaluating the questionnaire.

__________________________________________________________________________________

__________________________________________________________________________________

Is there anything else you would like to tell us about digital health technologies?

__________________________________________________________________________________

__________________________________________________________________________________

__________________________________________________________________________________

Thank you for taking the time to complete our questionnaire!

Even if you do NOT want to take part in the “Virtual Assisted Living” project, we are interested in your opinion and would like to conduct an interview with you on the subject of digital health technologies*.

** A total of 10 personal interviews are planned as part of the survey on health technologies by employees of Charité – Universitätsmedizin Berlin.*

**S5. Survey for participants**

# **Questionnaire in the Virtual Assisted Living project***

Content

[**Questionnaire in the Virtual Assisted Living project*** 9](#_Toc181369293)

[1 Current living situation 10](#_Toc181369294)

[2 Health related quality of life 10](#_Toc181369295)

[3 Control beliefs 10](#_Toc181369296)

[4 Fear of falling 11](#_Toc181369297)

[5 Perceived stress in daily life 11](#_Toc181369298)

[6 Social relationships 11](#_Toc181369299)

[6.1 Loneliness 11](#_Toc181369300)

[6.2 Sozial support 11](#_Toc181369301)

[7 Health related anxiety 11](#_Toc181369302)

[8 Competences to cope with daily life 11](#_Toc181369303)

[8.1 Activities of daily living 11](#_Toc181369304)

[8.2 Instrumental activities of daily living 11](#_Toc181369305)

[9 Technology 11](#_Toc181369306)

[9.1 Technology commitment 11](#_Toc181369307)

[9.2 Technology acceptance 11](#_Toc181369308)

[10 Sociodemographics 11](#_Toc181369309)

[11 Care and support 12](#_Toc181369310)

[12 Health 14](#_Toc181369311)

[13 Medication 16](#_Toc181369312)

[14 Reasons for participating in the study 17](#_Toc181369313)

[15 Survey framework 17](#_Toc181369314)

[16 Corona-Pandemic 18](#_Toc181369315)

[17 Depression screener 18](#_Toc181369316)

*Validated scales are only named together with their citation since we do not own the rights to copy the entire content of the questions. All self-devised scales are presented in their translated form.

# 1 Current living situation

**1.1 Who do you currently live with in this apartment?**

⭘ alone

⭘ with (spouse/life) partner

⭘ with daughter/son, stepdaughter/son, adopted daughter/son

⭘ with grandchild(ren

⭘ with daughter/son-in-law

⭘ with sister/brother

⭘ with other persons: ________________________________

**1.2 How many people live in your household in total, including yourself?**

____­­­_______ persons

**1.3 How long have you lived in this apartment?**

**since** (year) __ __ __ __

**1.4 Do animals live in your household?**

⭘ No

⭘ yes, which kind (and number)? ______________________________

**1.5 Is your apartment accessible by elevator?**

⭘ no ⭘ yes ⭘ partly

**1.6 On which floor is your apartment located?**

________. floor

**1.7 How many rooms does your apartment have?**

________ rooms

**1.8 postal code:** __ __ __ __ __

# 2 Health related quality of life

*SF-12 (Bullinger 1995, Bullinger et al 1998)*

# 3 Control beliefs

*1-Item*

Now it's about your perceived scope for action in relation to your health. Please select the answer option that applies to you.

| 1. | My physical health is largely dependent on what I do. | | | | |
| --- | --- | --- | --- | --- | --- |
|  | **Not true at all**  **1** | **Rather not true**  **2** | **Partly**  **3** | **Rather true**  **4** | **Completely true**  **5** |
|  | ⭘ | ⭘ | ⭘ | ⭘ | ⭘ |

# 4 Fear of falling

*FES-I (Falls Efficacy Scale – International Version) - Yardley, L., Beyer, N., Hauer, K., Kempen, G., Piot-Ziegler, C., & Todd, C. (2005). Development and initial validation of the Falls Efficacy Scale-International (FES-I). Age and ageing, 34(6), 614-619.*

# 5 Perceived stress in daily life

*Perceived Stress Scale German PSS-10 questionnaire - Schneider, E. E., Schönfelder, S., Domke-Wolf, M., & Wessa, M. (2020). Measuring stress in clinical and nonclinical subjects using a German adaptation of the Perceived Stress Scale. International Journal of Clinical and Health Psychology, 20(2), 173-181.*

# 6 Social relationships

# 6.1 Loneliness

*Loneliness Scale - De Jong-Gierveld, J., & van Tilburg, T. G. (2006). A 6-item scale for overall, emotional, and social loneliness: Confirmatory tests on survey data. Research on aging, 28(5), 582-598.*

# 6.2 Sozial support

*F-SozU K-14* (Fydrich, Sommer & Brähler, 2007)

# 7 Health related anxiety

*Modified short form of the Health Anxiety Inventory - MK-HAI - Bailer, J., Rist, F., Müller, T., Mier, D., Diener, C., Ofer, J., ... & Witthöft, M. (2013). Erfassung von Krankheitsangst mit dem short health anxiety inventory (SHAI). Verhaltenstherapie & Verhaltensmedizin, 34(4), 378-398.*

# 8 Competences to cope with daily life

# 8.1 Activities of daily living

*Barthel-Index - Mahoney, F. I., & Barthel, D. W. (1965). Functional evaluation: the Barthel Index: a simple index of independence useful in scoring improvement in the rehabilitation of the chronically ill. Maryland state medical journal.*

# 8.2 Instrumental activities of daily living

*IADL (Lawton & Brody, 1969)*

# 9 Technology

# 9.1 Technology commitment

*Technikbereitschaft - Neyer, F. J., Felber, J., & Gebhardt, C. (2012). Entwicklung und Validierung einer Kurzskala zur Erfassung von Technikbereitschaft. Diagnostica.*

# 9.2 Technology acceptance

*Reduced version of the* s*imple scale for acceptance measurement - Van Der Laan, J. D., Heino, A., & De Waard, D. (1997). A simple procedure for the assessment of acceptance of advanced transport telematics. Transportation Research Part C: Emerging Technologies, 5(1), 1-10.*

Please assess the home emergency call system with automatic fall detection and the sensors in the home.

|  | **1** | **2** | **3** | **4** | **5** |  |
| --- | --- | --- | --- | --- | --- | --- |
| useful | ⭘ | ⭘ | ⭘ | ⭘ | ⭘ | useless |
|  |  |  |  |  |  |  |
| assisting | ⭘ | ⭘ | ⭘ | ⭘ | ⭘ | worthless |

# 10 Sociodemographics

**10.1 Date of birth:** (D/M/Y) ____ . ____ . ________

**10.2 What is your gender?**

⭘ male

⭘ female

⭘ diverse

**10.3 What is your highest general school-leaving qualification?**

□ Finished school without a school-leaving certificate
□ Secondary or elementary school certificate
□ Secondary school leaving certificate/intermediate school leaving certificate/technical school leaving certificate
□ POS (polytechnic secondary school) or 10th grade (before 1965: 8th grade)
□ Technical college entrance qualification/graduation from a technical college
□ Abitur/general or subject-specific higher education entrance qualification
□ Other school-leaving qualification (e.g. obtained abroad)

**10.4 What is your highest vocational qualification?**

□ No vocational qualification
□ Apprenticeship (vocational/in-company training)
□ Training at vocational school/trade school (vocational-school training)
□ Technical school (master craftsman, technical school, vocational or technical academy)
□ University of applied sciences/engineering school
□ University or college
□ Other: ___________________________________________

**10.5 What is your current family situation?**

⭘ in partnership/marriage

⭘ single

**10.6** **How many children do you have?** __________ Child(ren)

**10.7 What is the best way to describe your personal financial situation?**

⭘ very good

⭘ good

⭘ average

⭘ difficult

⭘ very difficult

# 11 Care and support

**11.1 In which areas of daily life do you currently receive support?**

⭘ Taking medication

⭘ Leisure activities

⭘ Household

⭘ Body care

⭘ Organizational activities

⭘ Asset management

⭘ Accompanying to appointments

⭘ other: _______________________________

⭘ None

**11.2** **Which of the following aids do you currently use in your everyday life?**

⭘ Walking stick

⭘ Forearm crutch

⭘ Rollator

⭘ Walking frame

⭘ Manual wheelchair

⭘ Electric wheelchair

⭘ Visual aid

⭘ Hearing aids

⭘ Orthopaedic shoes/insoles/orthoses

⭘ Arm/leg prosthesis

⭘ other: _______________________________

⭘ None

- 1. **Do you make use of an offer from SOPHIA, if so, how often?**

⭘ Daily ⭘ several times a week ⭘ 1x weekly

⭘ several times a month ⭘ 1x monthly ⭘ never

- 1. **Do you receive support from other people (e.g. with care, nursing)?**
- ***Relatives***

⭘ daily ⭘ several times a week ⭘ 1x weekly

⭘ several times a month ⭘ 1x monthly ⭘ never

- ***Friends / neighbours***

⭘ daily ⭘ several times a week ⭘ 1x weekly

⭘ several times a month ⭘ 1x monthly ⭘ never

- ***Outpatient nursing service***

⭘ daily ⭘ several times a week ⭘ 1x weekly

⭘ several times a month ⭘ 1x monthly ⭘ never

- ***Volunteers***

⭘ daily ⭘ several times a week ⭘ 1x weekly

⭘ several times a month ⭘ 1x monthly ⭘ never

- ***Other people / organisations*:** ______________________________________________________­­­­­­­­_________

⭘ daily ⭘ several times a week ⭘ 1x weekly

⭘ several times a month ⭘ 1x monthly ⭘ never

**11.5 Do you have a care level?**

⭘ 1 ⭘ 2 ⭘ 3 ⭘ 4 ⭘ 5 ⭘ no care level ⭘ I don‘t know

⭘ no, but has been applied ⭘ Upgrading has been applied for

- 1. **What rights of representation do other persons have on your behalf?**

⭘ Health care proxy ⭘ Care directive ⭘ General power of attorney ⭘ None

# 12 Health

**12.1 How often do you consume:**

- Alcohol

⭘ never

⭘ on rare occasions

⭘ several times a month

⭘ several times a week

⭘ daily (what and how much?__________________________ /daily)

- Nicotin

⭘ never

⭘ several times a month

⭘ several times a week

⭘ daily (what and how much? __________________________ /daily)

- Other drugs:

⭘ no

⭘ yes

If yes, which ones? ________________________________

**12.2 Which of the following illnesses/symptoms have you experienced in the last 12 months?**

⭘ Ischemic heart disease

⭘ Heart failure

⭘ other cardiovascular diseases
⭘ Bronchitis & COPD
⭘ Mental behavioral disorders due to alcohol and opioids
⭘ Back pain
⭘ Hypertension
⭘ Gastroenteritis and certain intestinal diseases
⭘ Influenza and pneumonia
⭘ Ear, nose and throat infections
⭘ Depressive disorders
⭘ Diabetes mellitus
⭘ Osteoarthritis of the knee
⭘ Diseases of the tendons and soft tissue
⭘ Diseases of the eye
⭘ Diseases of the urinary system
⭘ Sleep disorders
⭘ Infections of the skin and subcutaneous tissue
⭘ Malnutrition
⭘ Dental and oral cavity diseases
⭘ Apoplexy
⭘ Parkinson's syndrome
⭘ Dementia
⭘ Polyneuropathy
⭘ Myasthenia, muscle atrophy
⭘ Dizziness and vertigo
⭘ Epilepsy
⭘ Cancer
⭘ Other serious illnesses: _____________________________________

- 1. **How often have you been hospitalized in the last 12 months?**

____________ times

- 1. **Have you had pain within the last 24 hours?**

⭘ yes ⭘ no

**If yes, please indicate your average pain in the last 24 hours on a scale from 0 (no pain) to 10 (maximum imaginable pain):**

*Brief Pain Inventory (BPI) - Radbruch, L., Loick, G., Kiencke, P., Lindena, G., Sabatowski, R., Grond, S., ... & Cleeland, C. S. (1999). Validation of the German version of the Brief Pain Inventory. Journal of pain and symptom management, 18(3), 180-187.*

**12.5 Have you fallen, slipped, or tripped in the last 12 months, causing you to lose your balance and fall to the ground or some lower level?**

⭘ yes ⭘ no

**If yes, how often?** _____________ times

|  | **1. event** | **2. event** | **3. event** | **4. event** | **5. event** |
| --- | --- | --- | --- | --- | --- |
| **Where did the fall take place?** | | | | | |
| außerhalb der Wohnung | ⭘ | ⭘ | ⭘ | ⭘ | ⭘ |
| innerhalb der Wohnung | ⭘ | ⭘ | ⭘ | ⭘ | ⭘ |
| **If inside the apartment, where exactly?** | | | | | |
| Hallway | ⭘ | ⭘ | ⭘ | ⭘ | ⭘ |
| Bath | ⭘ | ⭘ | ⭘ | ⭘ | ⭘ |
| Kitchen | ⭘ | ⭘ | ⭘ | ⭘ | ⭘ |
| Living room | ⭘ | ⭘ | ⭘ | ⭘ | ⭘ |
| Bedroom | ⭘ | ⭘ | ⭘ | ⭘ | ⭘ |
| Other room | ⭘ | ⭘ | ⭘ | ⭘ | ⭘ |

- 1. **Manual force measurement**

Height: ______ cm

Weight: ______ kg

Hand circumference: ______ cm Size pressure ball: ⭘ mittel ⭘ groß

dominant Hand: ⭘ right ⭘ left

parethic Hand: ⭘ no ⭘ yes: __________

1. Measurement: right: __________bar / left: __________bar
2. Measurement: right: __________bar / left: __________bar
3. Measurement: right: __________bar / left: __________bar

# 13 Medication

**What medication are you currently taking? Please also indicate those that have not been prescribed by a doctor**

**(e.g. herbal preparations, vitamins etc.).**

| **Nr.** | **Active substance or trade name** | **Single dosing  (mg, ml …)** | **Dosage form**  **(Tbl., drops, ...)** | **Frequency of use**  **(1-0-1-0 or**  **if required)** | **with doctor's prescription / recommendation** | **without doctor's prescription / recommendation** |
| --- | --- | --- | --- | --- | --- | --- |
|  |  |  |  |  | ⭘ | ⭘ |
|  |  |  |  |  | ⭘ | ⭘ |
|  |  |  |  |  | ⭘ | ⭘ |
|  |  |  |  |  | ⭘ | ⭘ |
|  |  |  |  |  | ⭘ | ⭘ |
|  |  |  |  |  | ⭘ | ⭘ |
|  |  |  |  |  | ⭘ | ⭘ |
|  |  |  |  |  | ⭘ | ⭘ |
|  |  |  |  |  | ⭘ | ⭘ |
|  |  |  |  |  | ⭘ | ⭘ |

# 14 Reasons for participating in the study

**What prompted you to take part in this study?**

____________________________________________________________________________________________________________________________________________________________________

__________________________________________________________________________________

__________________________________________________________________________________

# 15 Survey framework

**Measurement point: ⭘ t0 ⭘ t1**

**Have you already installed the home emergency call system with automatic fall detection and the sensors in your home??**

- yes, before t0 (date: ____________)
- no, not yet

**Did you already have a home emergency call before participating in the Virtual Assisted Living project?**

- yes
- no

**How was the survey conducted?**

- Participant was interviewed in person
- The questionnaire was sent to the participant by post
- Participant was interviewed by telephone

**Was the survey terminated prematurely?**

- yes ⭘ no

**Comments on the survey/termination** *(special features in the course or in communication; presence and influence of third parties, problems with individual questions, reasons for termination ...)***:**

______________________________________________________________________________________________________________________________________________________________________________________________________________________________________________________

# 16 Corona-Pandemic

*Petzold, M. B., Bendau, A., Plag, J., Pyrkosch, L., Mascarell Maricic, L., Betzler, F., ... & Ströhle, A. (2020). Risk, resilience, psychological distress, and anxiety at the beginning of the COVID‐19 pandemic in Germany. Brain and behavior, 10(9), e01745.*

**Have you ever been diagnosed with COVID-19?**

⭘ no ⭘ yes

**Have you already had a corona test?**

⭘ no ⭘ yes

***If so, how often and with what results?*** ______ times (___ x positive/ ___ x negative)

**Have you already been vaccinated against coronavirus?**

⭘ no ⭘ yes

# 17 Depression screener

*PHQ-2 - Löwe, B., Kroenke, K., & Gräfe, K. (2005). Detecting and monitoring depression with a two-item questionnaire (PHQ-2). Journal of Psychosomatic Research, 58(2), 163-171.*
